# Knowledge, attitude, and ethical concepts of Sudanese medical students regarding stem cells and its application

**DOI:** 10.1097/MD.0000000000035768

**Published:** 2023-11-03

**Authors:** Lina Hemmeda, Esraa S.A. Alfadul, Mohamed Satti, Alaa S. Ahmed, Ammar Elgadi, Sara Emad, Lina Elfaki, Mohammed Mahmmoud Fadelallah Eljack

**Affiliations:** a Faculty of Medicine, University of Khartoum, Khartoum, Sudan; b Teaching Assistant, Faculty of Medicine, University of Bakht Alruda, Ad Duwaym, Sudan.

**Keywords:** attitude, ethics, knowledge, stem cells

## Abstract

One of the major challenges that stem cell transplantation faces is a lack of donors due to a lack of knowledge and awareness of the importance of stem cell transplantation, this implies that health care providers should arm themselves with sufficient knowledge to contribute positively to raising awareness. This is an analytical cross-sectional study of 1040 medical students from 10 universities from various Sudanese states, through an online self-administered pre-tested and structured questionnaire formulated by the authors with a particular focus and/or reflection on the knowledge and attitudes of medical students. The median knowledge score among all students was 8.0 (6–9) with the majority of students confirming that stem cells are capable of dividing and can self-renew for a long period (88.6%). Regarding attitude, the median score among the participants was 23 (17–27) with (47.9%) agreeing that competency in stem cell knowledge is important for them as future health care providers. In terms of ethical attitude; the majority of the students (59, 2%) think there’s a need to obtain ethical approval before conducting research. Moreover, (45.9%) of students believe that health practitioners have the right to use stem cells in treatments if those treatments have been scientifically proven to be effective on animals and on human cells in the laboratory. It is important to promote educational programs that inform medical students about the full range of possibilities offered by stem cell research. Furthermore, more studies is required to determine how society and religion affect medical students’ attitudes toward stem cells.

## 1. Introduction

In 1998, a scientist named Thomson isolated embryonic stem cells from fertilized eggs, firing a revolution in science known as stem cell research, which has now resulted in over 5000 registered clinical studies in the NIH database, and that number is growing.^[1]^

Stem cells are primitive “non-specialized” cells that divide and renew continuously during early life and growth to form a specific cell or tissue type.^[2,3]^ There are 2 types of stem cells: embryonic stem cells isolated from the inner mass of blastocysts and adult stem cells found on adult tissues.^[4,5]^ They are found in all multicellular organisms and can regenerate through mitotic cell division and differentiate into a variety of specialized cell types.^[4]^ They can be aspirated with a needle from the posterior iliac bone of a person of a suitable tissue type or the individuals themselves, and then given to the recipient after preparation; this is known as stem cell transplantation, a procedure that can save a life for patients with malignancy, autoimmune diseases, and organs failure.^[6,7]^

This has sparked high hopes and promises within scientific communities, as well as warnings and ethical, political, and religious debates about the destruction of embryonic human cells.^[6]^ These various points of view are strongly associated with opposing stem cell research, as President Bush did in 2001, or supporting it, as President Obama did in 2009, and thus occur in other countries.^[1]^ Later, scientists discovered alternatives to embryonic stem cell destruction, such as adult stem cells and pluripotent cells, which are largely similar to embryonic stem cells and are now used to develop insulin-producing cells with pancreatic cell characteristics, retinal cells for corneal transplants, and dopamine-secreting neurons.^[1]^

In Muslim countries, there is no legislation governing stem cell research.^[3]^ Saudi Arabia began performing transplants using cord blood (CB) units imported from international registries by the King Faisal Specialist Hospital and Research Center (KFSH-RC) in 2003, and as the Arab world’s first country, it recorded more than 10000 stem cells in 2015. Al-Neelain University established the Al-Neelain Stem Cell Center in December 2018 as Sudan’s first national stem cell facility.^[8]^ It conducts important stem cell research in Sudan, collaborates with other international stem cell research facilities, collaborates with educational and medical institutions to disseminate stem cell therapy techniques, and trains researchers at the center through workshops and seminars.^[8]^

One of the major challenges that stem cell transplantation faces is a lack of donors due to a lack of knowledge and awareness of the importance of stem cell transplantation among the general population.^[7]^ This implies that healthcare providers should arm themselves with sufficient knowledge to contribute positively to raising awareness and achieving the goal of making stem cell banking an integral part of healthcare.^[4]^ Medical students, as a group of young, healthy, future healthcare leaders who are open to new knowledge and easily accessible, will be sources of information or misinformation and may influence patient behavior via social norms.^[6,9]^ Despite these facts, medical students continue to be a particularly underserved population when it comes to assessing knowledge, attitudes, and behaviors regarding stem cell transplantation.^[7]^ Many studies have been conducted in various countries to assess the knowledge, attitudes, and practices of health professionals and students being educated in the field of health. Four studies from Saudi Arabia revealed inadequate levels among nurses and dentists, while physicians and medical students demonstrated a moderate level.^[2,3,6,7,10]^ Another study on Indian dentistry students revealed a lack of knowledge, whereas Malaysian nursing medical students demonstrated a moderate level of knowledge.^[11,12]^ When it comes to Nigerian dentistry medical students, demonstrated a high level of awareness and attitude toward stem cells but a lack of knowledge in their application in dentistry.^[5]^

Despite the breadth of studies undertaken in the region, there are no studies in Sudan that examine students’ knowledge, attitudes, and practices related to stem cells to the best of our knowledge. With the increased need for donors, it will be advantageous to first assess students’ knowledge, attitudes, and practices of students being taught health in terms of topic and secondary, to conduct education and courses on stem cells and its applicants. The purpose of this study is to determine Sudanese medical students’ knowledge, attitude, and practice regarding stem cells and to link this relationship to sociodemographic factors.

## 2. Methods

### 2.1. Design and settings

An analytical cross-sectional study was carried out between 15 June 2022 and first July 2022. All registered medical students from 10 Sudanese universities were included in the study. Those who refused to participate, as well as those who had recently relocated from outside Sudan, were excluded.

To better represent the students, we choose all public medical schools in Khartoum state, Sudan’s capital, where approximately 60% of all medical schools were found; however, outside of Khartoum state, we choose at random the largest public medical schools in the middle, eastern, northern, southern, and western Sudan states.^[13]^

Five of the universities chosen were in Khartoum state (the capital of Sudan): the University of Khartoum, the University of Alzaeim Alazhari, the University of Al-Neelain, the University of Bahri, and the Omdurman Islamic University. Accounts for 7000 medical students approximately.

Universities in non-capital states include the University of Gezira in Gezira State, the University of El-imam Elmahdi in White Nile State, the University of the Red Sea in Red Sea State, the University of Nile Valley in River Nile State, and the University of North Kordofan in North Kordofan State. Approximately 5000 students.

### 2.2. Sampling and participant

A total coverage of medical students from all academic years was included in the study. Those who refused to participate were excluded from the study. The sample size was calculated using the following formula X = *Z*^2 * *p* * (1 − *p*)/MOE^2. With a 95% confidence level and 0.05 margin of error, the calculated minimum sample size was 384 participants.

### 2.3. Instrument development

An online self-administered questionnaire was formulated by the authors based on an assessment of the literature on stem cells and their use in the field of medicine among medical students, with a particular focus and/or reflection on knowledge and attitudes.^[2,5–7,10–12]^

Students from each university had been recruited to collect the data and added as citable collaborators to this study. using online social media platforms (Facebook, WhatsApp, Instagram, Twitter…etc), data collection was started using Google form links. An introduction page contains information about the author’s details, research importance, and objective.

The questionnaire consists of 4 variables Section one: was the demographic data assessed via explanatory questions including sex, university affiliation, and year of study. Section 2: was the knowledge of stem cells including 17 dichotomous, which consist of either yes or no. Section 3: asked 11 questions on a 5-point Likert scale about students’ attitudes toward stem cells for medicinal applications. Each respondent was asked to choose between strongly disagreeing, disagreeing, being unsure, agreeing, or strongly agreeing with the statements presented to them.

One question was assessing the knowledge of the application of stem cells in medicine.

Finally, section 4 addresses stem cell ethics. Each question was scored on a 3-point Likert scale as yes, no, or unsure.

### 2.4. Validity and reliability of the instrument

To verify the precision and reliability of the instrument created by the researcher in this study, an expert panel assessed and confirmed the instrument’s content before pilot research was carried out among a chosen group of 30 students from various universities. Thirty respondents were chosen because a basic rule of thumb is to use 30 or larger as an estimation parameter; hence 30 respondents were selected for this pilot study. Results from the pilot study Elaborate (each section).

In the reliability test of Cronbach alpha, Knowledge (0.705), attitude (0.862), ethics (0.668), overall (0.813), By doing so, it was possible to show the instrument’s validity and reliability ratings while also giving the researcher some insightful information and serving as an internal review of the questionnaire.

There for, the likelihood of issues and problems during the data-gathering phase can be considerably reduced.

### 2.5. Ethical consideration

Ethical approval was obtained from the health sector ethical review committee, (No: 12-22), 24/3/2022, University of Gezira, Wad Medani, Sudan.

All respondents were required to sign a written consent form, confirming their desire to participate in the study with a sentence informing them that they can exist at any step if they decide not to complete it.

To ensure privacy and anonymity, their IP addresses were not collected.

## 3. Results

### 3.1. Demographics

A total of 1040 medical students participated in the main study. On average, the participants were predominantly female (65%). A number of 10 universities were selected with an approximate number of 104 participants from each university. The majority of the students were from the fourth (26.3), second (20.3%), and fifth years (20.1%) with an average of 173 students in each academic year.

### 3.2. Descriptive

#### 3.2.1. The total score.

For the 1040 students who participated in this study, the overall median total score was 34 (27.5–38.5).

### 3.3. Knowledge

The median knowledge score among all students was 8.0 (6–9). The majority of students confirmed that stem cells are capable of dividing and can self-renew for a long period (88.6%); correctly stated that Stem cells can be used to test new drugs and their effectiveness (76.1%); knew that embryonic stem cells are capable of forming any cell type in the body (75.9%).

However, we detected a lack of awareness in some aspects; for example, only (54.6%) of students recognize that Sperm and eggs are a source for adult stem cells; (61.7%) knew that embryonic stem cell transplantation has a serious disadvantage as it could results in tumor formation, and not more than (63.6%) of students admitted that autologous adult stem cell transplantation is controversial primarily because of the immunogenic graft rejection.

In terms of stem cell applications, the majority agreed that stem cells can be used to test new drugs and their effectiveness, and that stem cells can be used to treat Parkinson’s, Alzheimer’s, cancer, diabetes, or heart diseases (76.1%) and (77.4%) respectively (Table 1).

**Table 1:** Knowledge, attitude, and ethical attitude summary.

Knowledge						
Question	Yes				No	
	N	%			N	%
Stem cells can be used to treat Parkinson’s, Alzheimer’s, cancer, diabetes or heart diseases	807	77.4%			235	22.6%
14. Stem cells can be induced from normal skin cells by switching on genes controlling the pluripotent and differential of stem cells	675	64.9%			365	35.1%
2. Stem cells are capable of dividing and can self-renew for long periods	921	88.6%			119	11.4%
12. Embryonic stem cell transplantation has a serious disadvantage as it could result in the formation of tumors	642	61.7%			398	38.3%
13. Umbilical cord blood stem cell transplantation is less efficient compared with bone marrow stem cell transplantation	682	65.6%			358	34.4%
5. A blastocyst should be given the same respect and right to live as a living human adult	568	54.6%			472	45.4%
10. There should be more awareness program regarding stem cell	661	63.6%			379	36.4%
9. Umbilical cord blood stem cell transplantation has a lower risk for graft versus host disease than other types of stem cells	675	64.9%			365	35.1%
Stem cells can be used to test new drugs and its effectiveness	791	76.1%			249	23.9%
6. Embryonic stem cells are capable of forming any cell type in the body including placenta	789	75.9%			251	24.1%
7. Umbilical cord blood stem cells are embryonic stem cells	773	74.3%			267	25.7%
**Attitude**						
Question	Strongly disagree	disagree	Neutral		Agree	Strongly Agree
11. The future of mankind is bright if stem cell research could be successfully conducted.	121 (11.6%)	143 (13.8%)	277 (26.6%)		270 (26.0%)	229 (22.0%)
10. There should be more awareness program regarding stem cell	128 (12.3%)	121 (11.6%)	237 (22.8%)		271 (26.1%)	283 (27.2%)
7. I would advise pregnant mothers to store their umbilical cord blood stem cells for future purposes	160 (15.4%)	228 (21.9%)	332 (31.9%)		207 (19.9%)	113 (10.9%)
8. Competency in stem cell k0wledge is important for me as a health care provider	123 (11.8%)	141 (13.6%)	277 (26.6%)		301 (28.9%)	198 (19.0%)
5. A blastocyst should be given the same respect and right to live as a living human adult.	151 (14.5%)	167 (16.1%)	346 (33.3%)		226 (21.7%)	150 (14.4%)
2. I am worried that stem cell transplantation might potentially open doors to human being killed for the benefit of others	170 (16.3)	283 (27.2%)	326 (31.3%)		190 (18.3%)	71 (6.8%)
4. Life begins at conception; thus, embryonic stem cell research which involves the destruction of embryo is immoral, illegal and unnecessary	168 (16.2%)	226 (21.7%)	296 (28.5%)		191 (18.4)	159 (15.3%)
**Ethical attitude**						
Question		No		Not sure		Yes
3. Patients may be treated with stem cells if the patient agrees		100 (9.6%)		264 (25.4%)		676 (65.0%)
7. Health practitioners have the right to use stem cells in treatments if those treatments have been scientifically proven to be effective on animals and on human cells in laboratory		195 (18.8%)		368 (35.4%)		477 (45.9%)
4. There is a need to obtain ethical approval to test cell treatments on animals		528 (50.8%)		314 (30.2%)		198 (19.0%)
11. I think stem cell therapy is a kind of alternative medicine that does not fall under medical regulations		277 (26.6%)		427 (41.1%)		337 (32.3%)
10. Pharmaceutical companies hinder progress in stem cell research for fear of competition		142 (13.7%)		600 (57.7%)		298 (28.7%)
9. It is important to obtain ethical approval from an ethics committee before conducting research		145 (13.9%)		279 (26.8%)		616 (59.2%)

### 3.4. Attitude

Overall, the median score for attitude toward stem cells among the participants was 23 (17–27). All the participants (47.9%) agreed that competency in stem cell knowledge is important for them as future healthcare providers. In the end, the majority of the students (53,3%) agreed that there should be more awareness programs regarding stem cells.

However, almost (74.8%) of the students were unworried or unsure that stem cell transplantation might potentially open doors to human beings killed for the benefit of others. Moreover, percentages of (66.4%) of the students disagreed or were unsure that embryonic stem cell which involves the destruction of an embryo is immortal, illegal, and unnecessary. In addition, (63.9%) of the students disagreed or were unsure that blastocysts should be given the same right and respect to live as a living human adult (Table 1).

### 3.5. Ethical attitude

The median score for ethical attitude towards stem cells and their application among the students was 3.5 (3–4.5). Regarding the ethical attitude of the students; the majority of the students (59, 2%) think there’s a need to obtain ethical approval before conducting research respectively. Moreover, (45.9%) of students believe that Health practitioners have the right to use stem cells in treatments if those treatments have been scientifically proven to be effective on animals and human cells in the laboratory.

On the other side, (50.8%) think there’s no need to obtain ethical approval to test cell treatments on animals, and (57,7%) were not sure whether or not pharmaceutical companies hinder progress in stem cell research for fear of competition (Table 1).

### 3.6. Statistical tests and correlations

#### 3.6.1. Knowledge, attitude, and ethical attitude across universities.

we compared the median scores of the universities individually (Table 2). A Kruskal–Wallis Test revealed a statistically non-significant difference in knowledge levels across different universities, χ^2^ (9, n = 1040) = 14.1, *P* = .119.

**Table 2:** Knowledge, attitude, and ethical attitude across universities.

	Knowledge (Md)	Attitude (Md)	Ethics (Md)	Total score (Md)
Alzaeim Alazhari	8.00	21.00	3.50	31.50
University of Bahri	8.00	21.50	3.50	32.50
University of El-imam Elmahdi	8.00	23.00	3.50	34.00
University of Gezira	7.00	21.00	4.00	32.50
University of Khartoum	8.00	23.00	3.50	35.00
University of Kordofan	8.00	25.00	3.50	37.00
University of Al-Neelain	8.00	25.00	4.00	36.00
University of Nile Valley	8.00	22.00	3.50	33.00
University of Omdurman	8.00	23.50	3.50	34.50
University of red sea	7.50	21.00	3.50	31.50
Total	8.00	23.00	3.50	34.00
H	14.096*^,^†	29.089*	20.616*	31.342*
*P* value	.119	.001	.014	<.001

* The test statistic is adjusted for ties.

† Multiple comparisons are not performed because the overall test does not show significant differences across samples.

However, Kruskal–Wallis test revealed a statistically significant difference in attitude levels across different universities, χ^2^ (9, n = 1040) = 29.1, *P* = .001. Pairwise tests were carried out for all 45 pairs of universities. There was strong evidence (adjusted using the Bonferroni correction) of a difference in attitude score between Alzaeim Alazhari and the University of Kordofan and the University of Al-Neelain. Students in the former have lower attitude scores compared to the latter 2 universities. There was no evidence of a difference between the other pairs.

Although a significant difference in ethical attitude levels across different universities, χ^2^ (9, n = 1040) = 20.62, *P* = .014, the pairwise test didn’t reveal any difference between the pairs suggesting that the significant *P* value represents a significant difference for a more complicated difference than that we have considered in the pairwise tests.

A significant difference in overall scores across different universities, χ^2^ (9, n = 1040) = 31.34, *P* ≤ .001. Pairwise tests were carried out for all 45 pairs of universities. There was strong evidence (adjusted using the Bonferroni correction) that Students at the University of Kordofan and the University of Al-Neelain had higher total scores compared to Alzaeim Alazhari students. However, the University of the red sea appears to have a significantly higher total score than the University of Kordofan and the University of Al-Neelain. There was no evidence of a difference between the other pairs.

### 3.7. Knowledge, attitude, and ethical attitude across gender

A Mann–Whitney U test revealed no significant difference in the knowledge levels of males (Md = 8, n = 364) and females (Md = 8, n = 676), U = 128200.5, i = 1.134, *P* = .257, *r* = 0.035; the attitude levels of males (Md = 22, n = 364) and females (Md = 23, n = 676), U = 125668, *z* = 0.571, *P* = .568, *r* = 0.018; the ethical attitude levels of males (Md = 3.5, n = 364) and females (Md = 3.5, n = 676), U = 116857.5, *z* = −1.351, *P* = .177, *r* = −0.041; and the attitude levels of males (Md = 33.25, n = 364) and females (Md = 34, n = 676), U = 126170, *z* = 0.679, *P* = .497, *r* = 0.021.

### 3.8. Correlations

The different relationships between Knowledge, attitude, and ethical attitude were investigated using Spearman’s rho correlation coefficient. There was a strong relation between ethical attitude and attitude, positive correlation between the 2 variables, *r* = 0.309, n = 1040, *P* < .001, with high levels of attitude associated with higher levels of ethical attitude. All the other correlations were positive but none of them reach a significant level (Table 3).

**Table 3:** Knowledge, attitude, and ethical attitude correlations.

	rs (*P*)		
	Knowledge	Attitude	Ethics
Knowledge		0.034 (.280)	
Attitude			0.309 (<.001)*
Ethical attitude	0.057 (.068)		

* Correlation is significant at the 0.01 level (2-tailed).

## 4. Discussion

Stem cell researches and applications are relatively new concepts that haven’t been fully utilized in Sudan and therefore this study aimed at getting an overview of the knowledge and attitude of Sudanese medical students towards it.

Our results showed that a great majority of the participants were aware of the ability of stem cells to divide and self-renew for long periods (88.6%), and almost two-thirds of them agreed that stem cells could be produced by manipulating the genes of normal skin cells (64.9%) those results are similar to a study conducted in Pakistan among medical students.^[14]^ Meanwhile, two-thirds of our participants (65.6%) admitted that umbilical cord stem cell transplantation is less efficient compared with bone marrow transplantation, in a similar study conducted among healthcare providers only a third of them (31.2%) agreed to that.^[4]^ This difference may be attributed to the fact that our students are all medical students studying medicine compared to the diversity in the fields among the other population, consisting mainly of health care providers other than doctors who may be less knowledgeable about stem cells. Our participants were more mindful of the disadvantage of stem cell transplantation as it could result in tumor formation (61.7%) compared to similar studies in Pakistan and Malaysia with lower percentages.^[12,14]^ Moreover, more than half of the participants (63.6%) agreed that there should be more awareness programs about stem cells, which reflects a good desire to learn among the students.

Regarding the attitude of the students towards stem cells the majority of the students were not worried or unsure that stem cell transplantation might open doors to human being killed for others (74.8%), this result correspondent to a study conducted by Mashal Daud.^[14]^ However, less than half of our participants (47.9%) consider that competency in stem cell knowledge is important for them as healthcare providers. Meanwhile, in a study conducted among healthcare providers a great majority of them (85.6%) considered competency in stem cells to be important,^[4]^ this might be because the latter have more contact with the clinical practice and have witnessed great advances in the field of medicine that made them address the need to the knowledge. Moreover, when asking our participants if they were to encourage pregnant women to store umbilical cord blood stem cells for future purposes only third of them agreed to that (30.8%), which is a lower percentage compared to that reported by Alzahrani et al where two thirds agreed (66%). This discrepancy may be explained by the fact that healthcare workers are more accustomed to the applications of stem cells in their practice than medical students and hence are more confident in promoting the idea.^[1]^

Interestingly our results showed no significant association between students’ Knowledge and attitudes toward stem cells indicated by the correlation coefficient r = .034. This finding suggests that other factors influence the attitude other than knowledge such as social, cultural, and religious factors. Jee Leng Lye in his research reached a similar finding.^[12]^

While investigating the ethical attitude of the students the majority agreed that patients may be treated with stem cells if the patients agree (65%) and that obtaining ethical approval is important before conducting research (59.2%) these findings are aligned with the finding of Alzahrani et al.^[1]^ More than half of the students weren’t sure whether pharmaceutical companies hinder the development of stem cell research or not (57.7%) which isn’t surprising bearing in mind that most medical students are not deeply involved in the specifications of the pharmaceutical industry.

## 5. Conclusion

Our study revealed that the majority of medical students have favorable attitudes and good knowledge of stem cells and their uses. It is critical to support educational programs that educate medical students about the breadth of opportunities presented by stem cell research. More research is also needed to understand how society and religion influence medical students’ views on stem cells.

### 5.1. Strength and limitations

Our study is the first study to address this issue in Sudan. Our sample was relatively large and diverse as 10 universities were included, and half represented states other than the capital. it also included all of the academic levels. Yet the use of non-probability sampling may affect the generalizability of the results and the sole use of the online version of the questionnaire may result in selection bias. In addition, our study didn’t address some factors that might be important such as religion and culture.

### 5.2. Recommendations

Educational Programs among medical students about all the potentials of stem cell research are recommended to encourage further research in this area. Also, further research is needed regarding the influence of religion and society on the attitude of medical students toward stem cells.

## Collaborators

Nuseiba Abdallah Mohammed Yasein, Faculty of Medicine, University of Al-Neelain, Khartoum, Sudan, Roaa B. Albashir, Faculty of Medicine, Red Sea University, Portsudan, Sudan, ElShimaa Ammar Mhagoub Zaki, Faculty of Medicine, Omdurman Islamic University, Omdurman, Sudan, Tagwa Abdelazem Mukhtar Awadelkarem, Faculty of Medicine, University of Bahri, Khartoum, Sudan, Maab Mansoor Jadkream Abdallah, Faculty of Medicine, University of Kordofan, El-Obied, Sudan, Zinab Taha Ahmed Abdallah, Faculty of Medicine, University of Elimam El-mahdi, Kosti, Sudan, Maab Saideldin Mohammed Alzain, Faculty of Medicine, University of Alzaim Alazhari, Khartoum, Sudan, Israa Mutasim Abdalla Mohamed, Faculty of Medicine, University of Gezira, Wad Medani, Sudan, Almthani Hamza Abdalrheem, Faculty of Medicine, Nile Valey University, Atbra, Sudan.

## Author contributions

**Conceptualization:** Esraa S.A. Alfadul.

**Data curation:** Lina Hemmeda, Esraa S.A. Alfadul, Alaa S. Ahmed.

**Formal analysis:** Mohamed Satti, Ammar Elgadi.

**Investigation:** Lina Hemmeda, Esraa S.A. Alfadul, Mohamed Satti, Alaa S. Ahmed, Ammar Elgadi, Sara Emad, Lina Elfaki, Mohammed Mahmmoud Fadelallah Eljack.

**Methodology:** Lina Hemmeda, Esraa S.A. Alfadul, Mohamed Satti, Alaa S. Ahmed, Ammar Elgadi, Sara Emad, Lina Elfaki, Mohammed Mahmmoud Fadelallah Eljack.

**Supervision:** Lina Hemmeda.

**Writing – original draft:** Lina Hemmeda, Esraa S.A. Alfadul, Mohamed Satti, Alaa S. Ahmed, Ammar Elgadi, Mohammed Mahmmoud Fadelallah Eljack.

**Writing – review & editing:** Sara Emad, Lina Elfaki, Mohammed Mahmmoud Fadelallah Eljack.
